# Issue Information

**DOI:** 10.1002/hsr2.121

**Published:** 2020-03-08

## Abstract

No abstract is available for this article.

